# A new sauropodiform dinosaur with a ‘sauropodan’ skull from the Lower Jurassic Lufeng Formation of Yunnan Province, China

**DOI:** 10.1038/s41598-018-31874-9

**Published:** 2018-09-07

**Authors:** Qian-Nan Zhang, Hai-Lu You, Tao Wang, Sankar Chatterjee

**Affiliations:** 1 Key Laboratory of Vertebrate Evolution and Human Origins of Chinese Academy of Sciences, Institute of Vertebrate Paleontology and Paleoanthropology, Chinese Academy of Sciences, 142 Xizhimenwai Street, Beijing, 100044 P. R. China; 2 CAS Center for Excellence in Life and Paleoenvironment, 142 Xizhimenwai Street, Beijing, 100044 P. R. China; 3 University of Chinese Academy of Sciences, 19A Yüquan Road, Beijing, 100049 P. R. China; 4 Bureau of Land and Resources of Lufeng County, Yunnan Province, 651299 P. R. China; 5 Museum of Texas Tech University, Lubbock, TX 79409 USA

## Abstract

The Early Jurassic Lufeng Formation of Yunnan Province in southwestern China is one of the best fossil localities in the world for understanding the early radiation of sauropodomorph dinosaurs. It has yielded a rich assemblage of complete and three-dimensionally preserved skeletons of herbivorous dinosaurs that provide crucial morphological information for systematic and evolutionary studies. Here we describe a new taxon, *Yizhousaurus sunae* gen. et sp. nov., represented by a nearly complete skeleton with an exquisitely preserved skull and mandible. *Yizhousaurus* is distinguished from other non-sauropodan sauropodomorphs by a unique combination of plesiomorphic and apomorphic features, which increases our understanding of the anatomical variation on the relatively conservative ‘prosauropod’ cranial plan. Phylogenetic analysis resolves *Yizhousaurus* as a sauropodiform, showcasing a mosaic character suite combining plesiomorphic states in the postcranial skeleton with some more ‘sauropodan’-like features in the skull. Furthermore, *Yizhousaurus* is placed closer to the base of Sauropoda than other non-sauropodan sauropodomorphs currently known from the Lufeng Formation, adding another taxon to enrich the Lower Jurassic Lufeng dinosaur fauna.

## Introduction

Sauropodomorphs represent the first successful radiation of herbivorous dinosaurs and include the largest terrestrial animals that ever walked on the Earth. They originated in the Late Triassic and underwent great diversification during the Early Jurassic throughout most of Pangea. A major diverging event leading to Sauropoda is hypothesized to have occurred in the split between the clades of Massospondylidae and Sauropodiformes. Recent cladistic analyses^1–5^ show that the taxa closest to the root of Sauropodiformes are mostly recovered from the Lower Jurassic Lufeng Formation in Yunnan Province, China, including *Yunnanosaurus*, *Jingshanosaurus* and the newly reported *Xingxiulong*^6^, which indicates Lufeng as an important area to document the origin and evolution of sauropodiform dinosaurs.

The Lufeng Jurassic strata is a typical continental redbed sequence that is traditionally divided into ‘Lower’ and ‘Upper’ Lufeng formations on the basis of lithology and fauna^7–10^. Here we adopt the ‘Lufeng Formation’ proposed by Fang *et al*.^11^ in replacement of the ‘Lower Lufeng Formation’; the Lufeng Formation is subdivided into two lithologic units: the Shawan Member (lower Dull Purplish Beds) and the Zhangjiaao Member (upper Dark Red Beds). Sun *et al*.^9^ recognized two distinctive vertebrate faunas corresponding to these two members in the Lufeng Basin, respectively. Both faunas are dominated by abundant non-sauropodan sauropodomorph dinosaurs, but many of these Lufeng taxa are poorly known in detail or vaguely diagnosed; thus their validity and/or systematic position is inconsistent among different researchers^12–16^.

**Figure 1:**
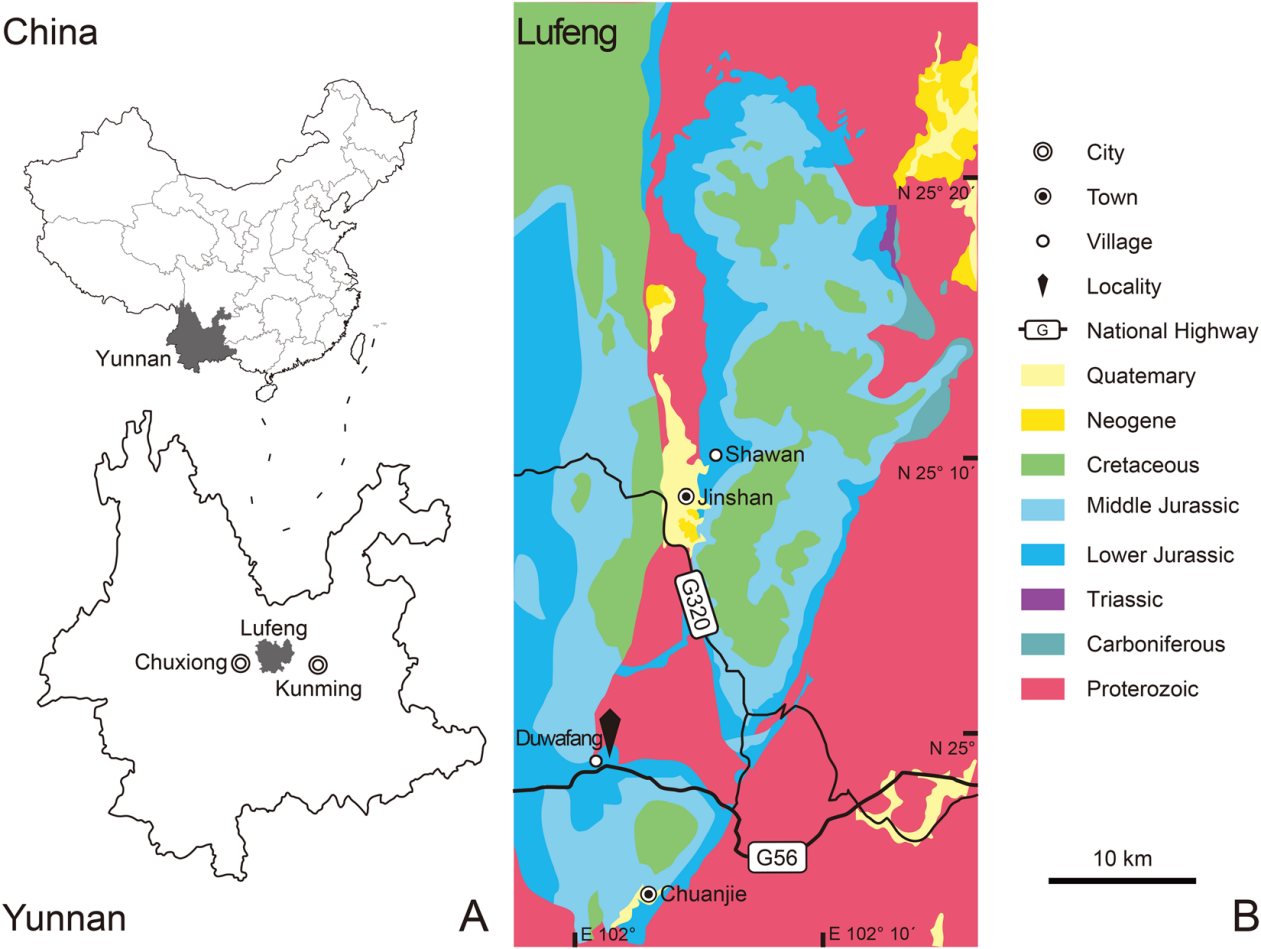
Geographic position and stratigraphic distribution of the locality of Yizhousaurus sunae gen. et sp. nov. (**A**) The location of Lufeng County in Yunnan Province, southwest China; (**B**), the geologic map of the area where *Yizhousaurus* was collected, the Lower Jurassic Lufeng Formation colored in marine blue. (These maps are created by Q.-N.Z using Adobe Illustrator CS5, http://www.adobe.com/cn/).

**Figure 2:**
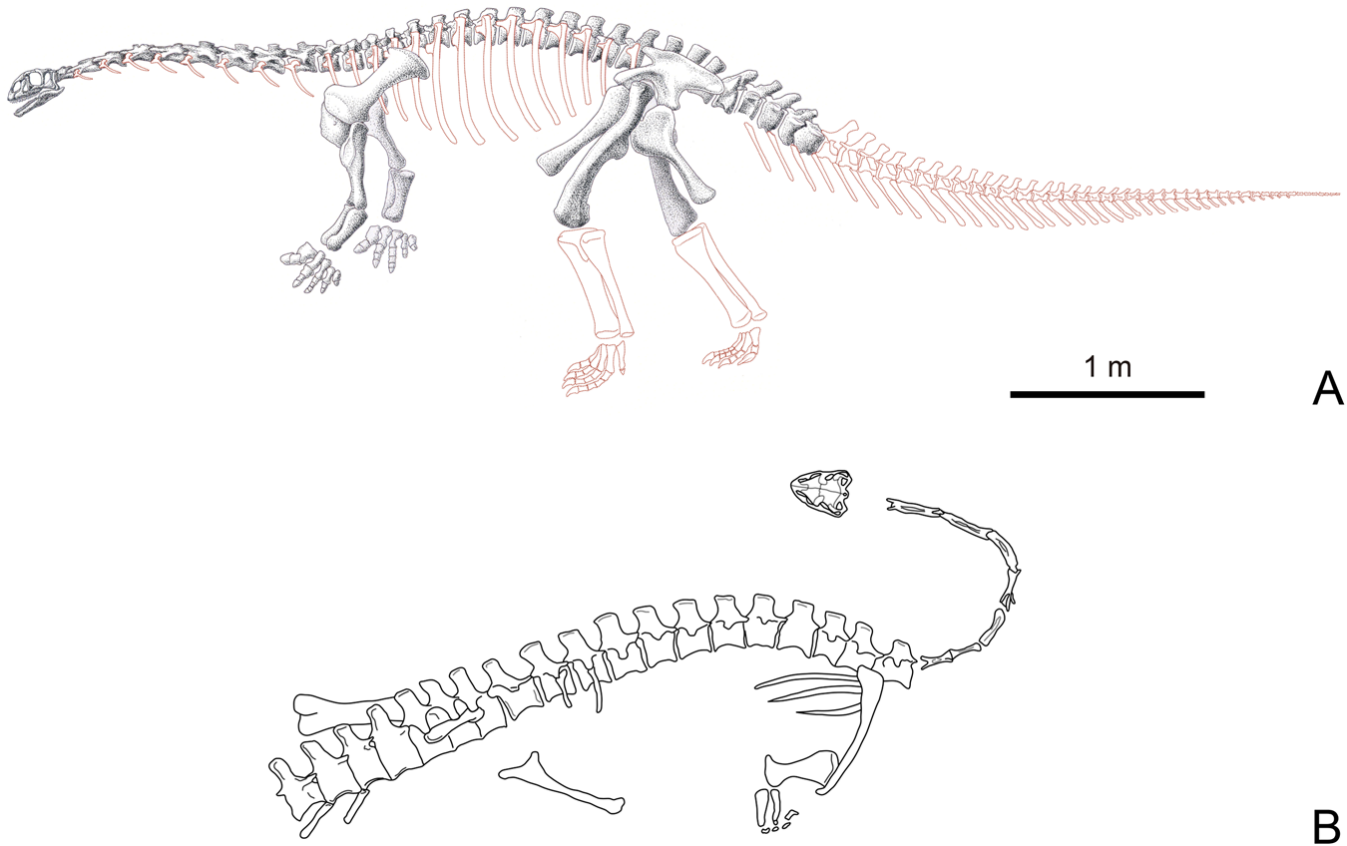
Status of preservation of Yizhousaurus sunae gen. et sp. nov. (**A**) The reconstruction in sketch of *Yizhousaurus* in left lateral view (drawn by Xiao-Cong Guo), regions in red rim represent absent elements; (**B**), the original burial map of *Yizhousaurus* (drawn by Q.-N.Z).

Here we describe a new sauropodiform taxon excavated from the Chuanjie Basin in Lufeng County, about 22 km south and lightly west to Shawan, which is one of the classic dinosaur localities in the Lufeng Basin (Fig. 1B). This specimen is represented by an articulated skeleton with an exquisite skull and mandible preserved in three-dimensions (Fig. 2B). Our phylogenetic analysis indicates that this new taxon is located in the heart of the sauropodiform-sauropod transformation, shedding new light on the radiation of sauropodiforms before the origin of sauropods.

## Results

Systematic Paleontology

Dinosauria Owen, 1842

Saurischia Seeley, 1887

Sauropodomorpha von Huene, 1932

Massopoda Yates, 2007

Sauropodiformes Sereno, 2007 (sensu^1^)

*Yizhousaurus sunae* gen. et sp. nov.

### Holotype

LFGT (Bureau of Land and Resources of Lufeng County, Yunnan, China) -ZLJ0033. An undistorted skeleton about 7 meters long, including a well-preserved skull and mandible, a mostly complete vertebral series (9 cervicals, 14 dorsals, 3 sacrals and 5 anterior caudals), pectoral and pelvic girdles, forelimbs (lacking both carpi) and both femora (Fig. 2A).

### Type locality and horizon

The specimen was collected near Duwafang Village, Chuanjie Town, Lufeng County, Chuxiong Yi Autonomous Prefecture, Yunnan Province, China (Fig. 1); the skeleton was excavated in the uppermost layer of the Zhangjiaao Member of the Lower Jurassic Lufeng Formation.

### Etymology

The generic name *Yizhou* refers to the Chuxiong Yi Autonomous Prefecture of Yunnan Province. The specific name is in honor of Professor Ai-Ling Sun, for her great contribution to Chinese vertebrate fossils, including those from Lufeng.

### Differential Diagnosis

A medium-sized sauropodiform distinguished from other non-sauropodan sauropodomorphs with respect to the following unique combination of character states (autapomorphies marked with *): lateral plates appressed to the labial sides of the premaxillary and maxillary teeth but not the dentary teeth*; anteroposterior expansion at the dorsal end of the maxillary ascending ramus; antorbital fenestra anteroposteriorly narrow and pipe-shaped in outline*; lacrimal shaft vertical with respect to the maxillary ramus*; transverse width of the ventral process of the postorbital greater than its anteroposterior width at midshaft; anterior tip of the dentary anterodosally curved over the alveolar margin*; tiny external mandibular fenestra (about 5% of the mandibular length)*; broad axial intercentrum wider than its centrum; deep depressions on the lateral surfaces of centra of dorsal vertebrae 3–6; hyposphenes of the anterior dorsals equal to their neural canals in height; and subelliptical cross-section of the midshaft of the femur.

### Comment

*Yizhousaurus sunae* was briefly reported as a basal sauropod at the Geological Society of America Conference in 2010, but has never received formal study. In addition, its fourth and fifth caudal vertebrae are fused together, which is considered pathological in nature^17^.

### Description

**Figure 3:**
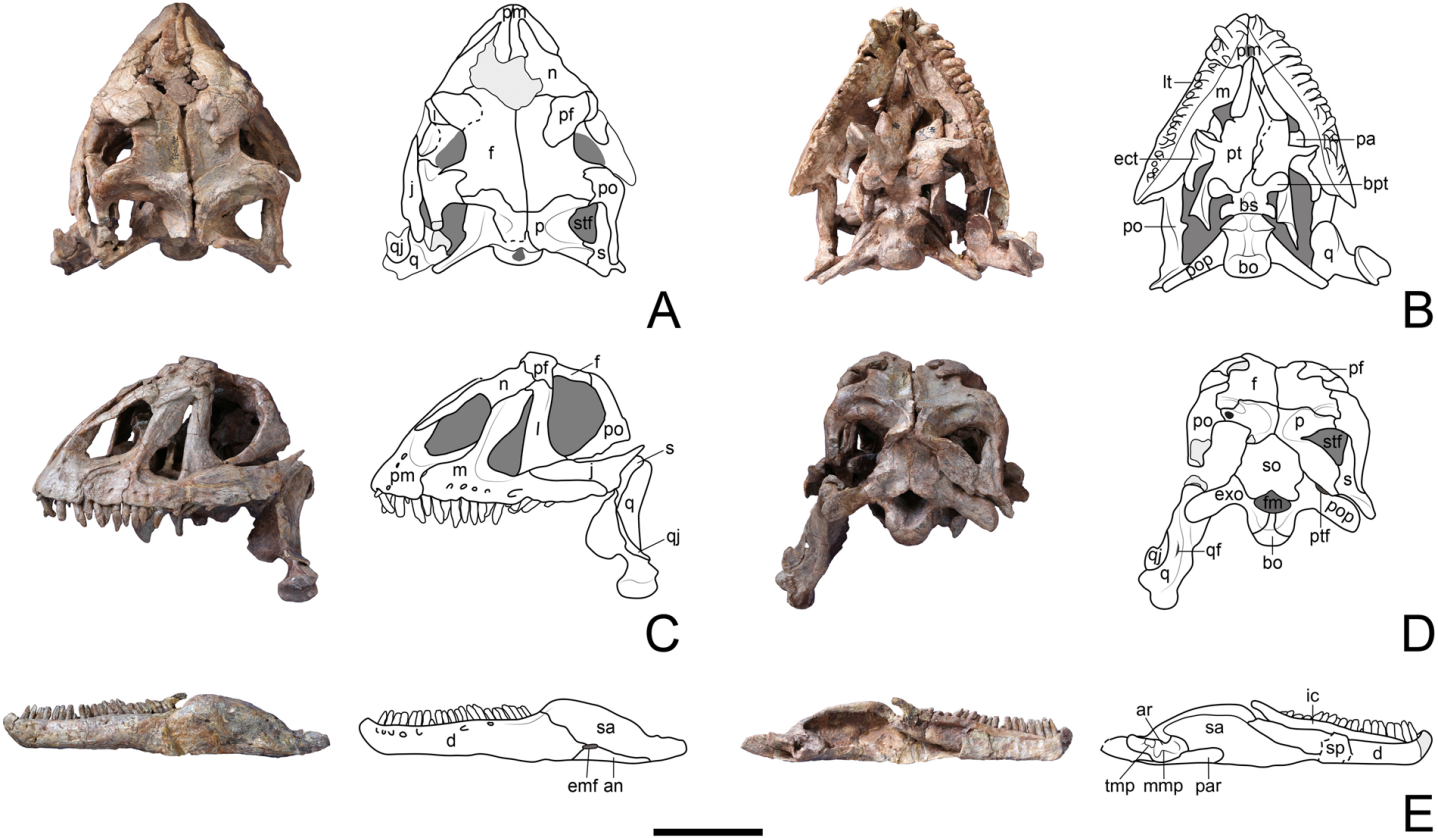
Skull and mandible of Yizhousaurus sunae gen. et sp. nov. (**A**–**D)** Photographs and interpretative line drawings of the skull in dorsal, ventral, left lateral and posterior views; (**E**), photograph and interpretative drawing of the left mandible in lateral and medial views. **Abbreviations:** an, angular; ar, articular; bo, basioccipital; bpt, basipterygoid process; bs, basisphenoid; d, dentary; ect, ectopterygoid; emf, external mandibular fenestra; exo, exoccipital-opisthotic complex; f, frontal; fm, foramen magnum; ic, intercoronoid; j, jugal; l, lacrimal; lt, lateral plate; m, maxilla; mpp, medial pyramidal process of the articular; n, nasal; p, parietal; pa, palatine; par, prearticular; pf, prefrontal; pm, premaxilla; po, postorbital; pop, paraoccipital process; pt, pterygoid; ptf, posttemporal fenestra; q, quadrate; qf, quadrate foramen; qj, quadratojugal; s, squamosal; sa, surangular; so, supraoccipital; sp, splenial; stf, supratemporal fenestra; tmp, tab-like medial process of the retroarticular process; v, vomer. Dark grey fills represent openings and light grey fills represent damage. Scale bar equals 10 cm. (The photographs are taken by Wei Gao, and line drawings are created by Q.-N.Z.)

The skull of LFGT-ZLJ0033 is almost complete, with a short snout, presenting a high and dome-shaped profile in lateral views. The skull has suffered a little compression lengthwise, so that it is slightly telescoped anteroposteriorly across the premaxilla-nasal suture; rotating the posterodorsal part of the skull roof clockwise, and pulling the ventral process of the postorbital in contact with the lacrimal. This has also resulted in partial damage to the quadrate, quadratojugal and squamosal. However, based on the well-preserved lower jaw (Fig. 3E), we can infer that the above deformation has only minimally affected the gross structural morphology of the skull. Most cranial openings of LFGT-ZLJ0033 are large and dorsoventrally elongated, whereas the antorbital fenestra is high but relatively narrow and appears pipe-shaped in lateral view (Fig. 3C), differing from the broad, crescentic or subtriangular fenestrae of most non-sauropodan sauropodomorphs, which can be considered as an autapomorphy of *Yizhousaurus*.

#### Skull roof

The most prominent feature of the premaxilla is the elongate nasal process that extends posterodorsally and slightly widens at the distal end (Fig. 3A), resembling the condition of several non-sauropodan sauropodomorphs (e.g., *Unaysaurus*^18^, *Plateosaurus*^19^, massospondylids^20,21^ and *Yunnanosaurus*^22^). The profile of the premaxilla is slightly convex without an inflection at the base of the dorsal process. On the lateral surface of the main body, at least three nutrient foramina are visible (Fig. 3C). The posterolateral process of the premaxilla extends posteriorly as a narrow process that overlaps the anterior process of the maxilla. The ventral tooth-bearing region of the premaxilla is robust and supports four teeth which are slightly longer than those of the maxilla. The lateral plates of the premaxilla extend ventrally to cover over half of the erupting teeth in ventral view (Fig. 3B). The horizontal main process of the maxilla extends posteriorly to receive the jugal on a dorsal shelf. The lateral maxillary ridge which is an autapomorphy of *Lufengosaurus*^16^ is absent on this process. A row of at least five neurovascular foramina are preserved just above the alveolar margins, and most of them are oriented anteroventrally, but the posterior-most one is oriented posteriorly and larger than the formers (Fig. 3C). The length of the maxillary anterior process is not greater than its dorsoventral depth, which is a feature sharing with *Yunnanosaurus*^22^, ‘*Melanorosaurus*’ (NMQR 3314)^23^ and a few massospondylids^20,24^, but contrary to the condition among most non-sauropodan sauropodomorphs. The ascending process of the maxilla is longitudinally high, and widens dorsally with a posteriorly projecting expansion (Fig. 3C), which is much pronounced than the same feature also seen in *Lufengosaurus*^16^ and *Adeopapposaurus*^20^. In ventral view, the lateral plates appressed to the labial side of maxilla are similar to those on the premaxilla, and extend around 1.5~2.0 cm, well below than the lingual alveolar margins; however, the lateral plates are absent on the dentary, unlike their presence in sauropods, and therefore this is regarded as a unique feature of *Yizhousaurus*. The maxillary tooth row terminates just below the middle of the orbit and bears 16 teeth. The marginal serrations are rarely preserved but are present on both mesiodistal sides in some teeth and restricted to the apical half of the crowns (see Supplementary Information). Both nasal bones are long and narrow, but their posterior regions are fractured with a large hole perforating the central parts of the skull roof (Fig. 3A). Each nasal gives rise to two processes, the anteroventral process is very short, ventrally extending for a limited distance along the ascending process of the maxilla (Fig. 3C), differing from the condition of most non-sauropodan sauropodomorphs, in which this process reaches approximately half of the maxillary ascending process. In *Plateosaurus*^19^ this process almost covers the entire ascending process and contacts the posterior process of the premaxilla, nevertheless, it extremely reduced in ‘*Melanorosaurus*’ (NMQR 3314)^23^ and sauropods. The lacrimal is inverted ‘L’-shaped in lateral view, consisting of a stout shaft almost perpendicular to the longitudinal axis of the maxilla, rather than anterodorsally sloping as in other non-sauropodan sauropodomorphs, and thus this feature is regarded as autapomorphic for *Yizhousaurus*. The dorsal process of the lacrimal is short and exposed in dorsal view, making a very small contribution to the skull roof (Fig. 3A). The prefrontal is strap-like and transversely convex in dorsal view, overlapping the frontal via a broad shelf. The ventrolateral process of the prefrontal clasps the posterodorsal corner of the lacrimal tightly and the ventromedial process extends along the lacrimal medially. The frontal is a broad, flat plate-like element, with the slender anterior process intruding between the prefrontal and nasal. The posterior end of the frontal is excluded from the anterior margin of the supratemporal fenestra by the parietal and the postorbital (Fig. 3A,D). The parietal consists of a central portion and two laterally directed processes; the anterolateral process is laterally curved and is shorter than the posterolateral one. The anterolateral process and the posterolateral process contact the frontal, postorbital, and the squamosal, respectively, together forming the medial floor of the supratemporal fossa. There is a tiny boss projected close to the junction of the parietal, postorbital and frontal (Fig. 3D), which is smaller than that seen in *Lufengosaurus*^16^. The postorbital is a triradiate bone, with the ventral process the longest of its three processes. It is subtriangular in cross section, with the mediolateral width of its midshaft wider than the anteroposterior length, sharing the condition with *Leyesaurus*^24^ and *Anchisaurus*^25^. Both jugals are broken posteriorly. The maxillary process of the jugal tapers anteriorly and inserts between the maxilla and lacrimal, but makes no contribution to the antorbital fenestra (Fig. 3C). Only the posteroventral corner of the left quadratojugal is preserved. It is a subtriangular lamina attached to the lateral surface of the quadrate and contacts the ventral tip of the squamosal. Both squamosals are poorly preserved, the left one only preserves its ventral process which has a subtriangular cross section, whereas the anterior process of the right one is overlapped by the posterodorsal process of the postorbital laterally (Fig. 3C,D).

#### Braincase

The supraoccipital has a pentagonal profile on the whole. It contacts the parietal dorsolaterally and the exoccipital-opisthotic complex ventrolaterally, together forming the borders of the posttemporal fenestra (Fig. 3D). The ventral margin of the supraoccipital arches to form the dorsal margin of the foramen magnum. The exoccipital-opisthotic complex is formed by the closely fused exoccipital and opisthtic, and ventrolaterally projecting the paroccipital process which is ended by a blunt tip. Each exoccipital contacts the ventrolateral margin of the supraoccipital and the dorsal region of the basioccipital, forming the lateral margin of the foramen magnum. The basioccipital expands posteriorly to form the occipital condyle that is sub-crescentic in posterior view. It constricts anteriorly to form a short neck, and then expands anteriorly to merge into the basal tubera with the basisphenoid (Fig. 3B). The basisphenoid floors the anterior region of the braincase. Its ventral surface is excavated with a wide median fossa. The basipterygoid processes are short rod-like elements, posterolaterally inclined and articulated with the pterygoids.

#### Quadrate and Palatal complex

The quadrate comprises a robust main shaft that receives the squamosal dorsally and the quadratojugal anteriorly. The ventral end of the shaft is expanded and divided into two condyles that articulate with the mandible. The medial condyle is larger and situated lower than the lateral one. There is an excavation delimited by a low ridge and incised into the body of the left quadrate (Fig. 3D), which is interpreted to be the quadrate foramen, as opposed to a cracked foramen on the quadrate-quadratojugal suture. The vomers are straight and elongate in ventral view; they are not parallel to each other due to displacement (Fig. 3B). The palatines are largely obscured by the pterygoids. The lateral process of the palatine is a sheet-like element that receives the medial surface of the maxilla. The pterygoids make up most of the palate, with its palatine process tapering anteriorly. The transverse flanges are ventrally expanded and laterally thickened to contact the ectopterygoids. Behind the central part of the pterygoid is a distinct socket formed by the medial process of the pterygoid to contain the basipterygoid process. The ectopterygoid is ventrally curved with its anteromedial process relatively thick, but the posterolateral process is small and short, projecting a tapering distally recurved tip against the medial surface of the jugal (Fig. 3B).

#### Mandible

The mandibular rami are slender and well-preserved. The external mandibular fenestra is clearly reduced, with the longest diameter about 5% of the total mandibular length (Fig. 3E), which can be considered as an autapomorphy of *Yizhousaurus*. Although, ‘*Melanorosaurus*’ (NMQR 3314)^23^, also possesses a small external mandibular fenestra, that of *Yizhousaurus* is considerably smaller than other non-sauropodan taxa. It should be noted that the borders of the external mandibular fenestrae of both *Jingshanosaurus*^26^ and *Chuxiongosaurus*^27^ are obscured by deformation and glue (pers. observ.). The elongate dentary occupies approximately two-thirds of the total mandibular length. Its anterior tip curves anterodosally over the alveolar margin, which is straight or weakly developed in other non-sauropodan sauropodomorphs, thus this feature is regarded as autapomorphic for *Yizhousaurus*. Several nutrient foramina are visible ventral to the alveolar margin on the lateral surface of the dentary. The dentition extends along most of the dorsal margin of the dentary, where it supports 23 close-packed teeth on the left, and 24 on the right. The surangular is gently sigmoid in outline, and the dorsal margin expands into an inward curled edge forming the dorsal margin of the coronoid eminence. It decreases in height posteriorly till the region of the jaw articulation, and tapers to join the formation of the retroarticular process. The angular is a strap-like element that forms the posteroventral region of the mandible, and is overlapped by the prearticular in medial view. The intercoronoid is a long and thin bony plate on the medial surface of the mandible. It is approximately 6 mm high and covers the lingual alveolar of the dentary from the third tooth to the last (Fig. 3E). Only a patch of the splenial is present on the medial surface of the left mandibular ramus, as a laminar bone appressed to the lingual dentary. The prearticular lacks its anterior portion, and tapers posteriorly as a subtrapeziform process attaching to the articular ventrally. The articular is irregular in shape, the dorsal surface of the anterior region is concave, and bears a pyramidal process medially. The posterior portion of the articular has a blunt tip and a small tab-like process extends from its medial surface (Fig. 3E), which has been described in *Coloradisaurus*^21^, and is also seen in some Lufeng specimens, such as *Xingxiulong*^6^, *Chuxiongosaurus*^27^ and *Jingshanosaurus* (pers. observ.).

#### Dentition

Most of the teeth are preserved but broken apically. They are lanceolate and labiolingually compressed, with basally constricted tooth crowns. The premaxillary and maxillary teeth are almost perpendicular to the maxilla, in addition the teeth of the dentaries are vertically oriented in the anterior half region, whereas inclined or recumbent in the posterior half. The labial surface of the tooth crown is slightly convex, and the lingual surface is almost flat. The crowns are apicobasally longer than mesiodistally wide, and the enamel is mostly smooth but bears gracile, longitudinal striations. Both the mesial and distal margins are serrated with denticles; the preserved serrations are all restricted to the apical half of the crowns, which can be observed on the emerging teeth of the maxilla and dentary.

#### Axial Skeleton

The vertebral column of LFGT-ZLJ0033 is well-preserved and contains a continuous series of nine cervicals, 14 dorsals, three fused sacrals and five anterior caudal vertebrae.

**Figure 4:**
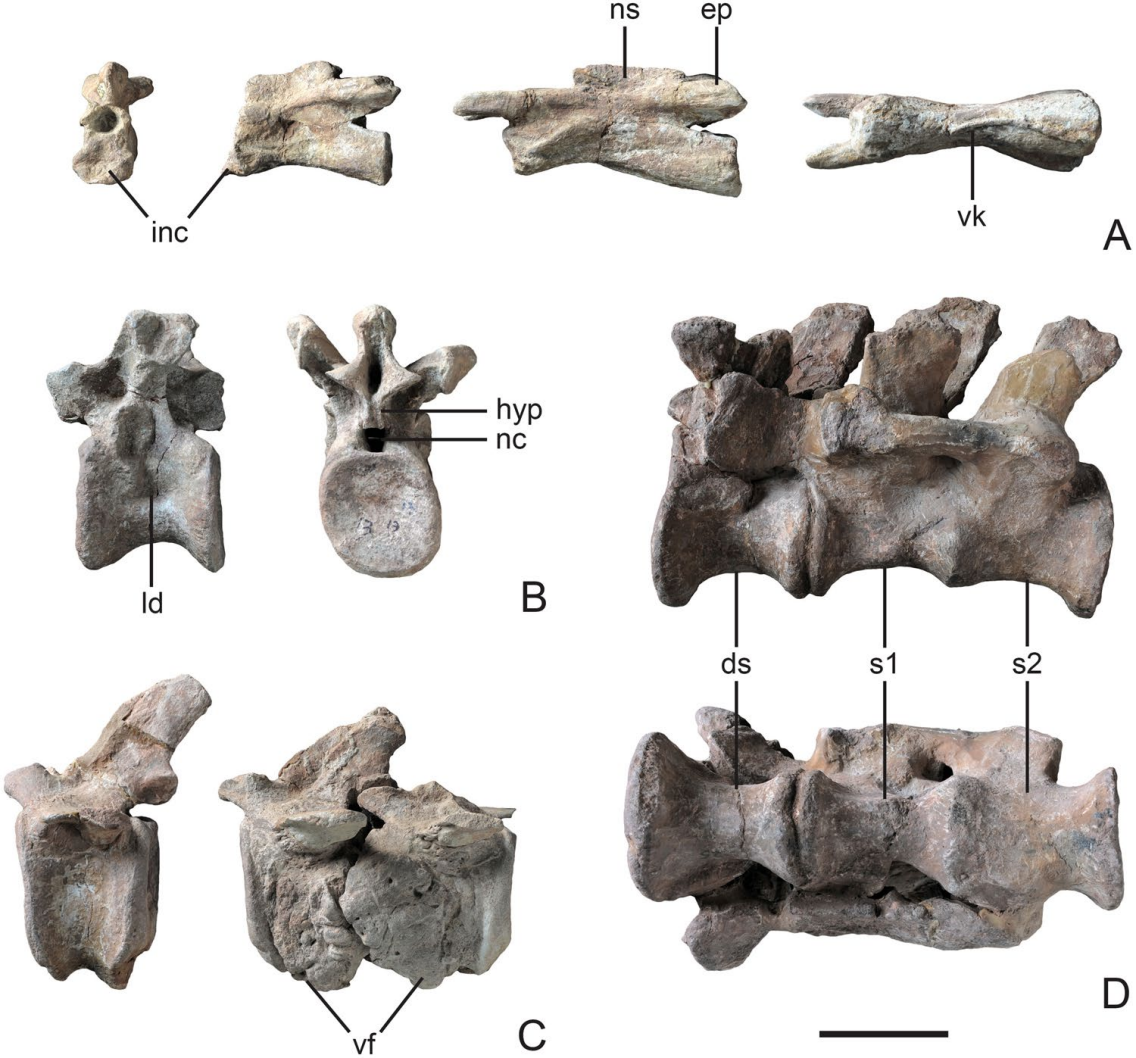
Vertebrae of Yizhousaurus sunae gen. et sp. nov. (**A**) Cervical vertebrae (axis in anterior and left lateral views, Ce3 in left lateral and ventral views); (**B**), dorsal vertebrae (D4 in left lateral and posterior view); (**C**), caudal vertebrae (Ca3-5 in left lateral view); (**D**), sacral vertebrae in left lateral and ventral view. **Abbreviations:** ds, dorsosacral; ep, epipophyses; hyp, hyposphene; inc, intercentrum; ld, lateral depression; nc, neural canal; ns, neural spine; s1, first primordial sacral; s2, second primordial sacral; vf, vertebral fusion; vk, ventral keel. Scale bar equals 10 cm. (The photographs are taken by Wei Gao.)

The centrum of the axis is more than three times longer than it is high. Its anterior articular surface presents a depression at its dorsal half, and the ventral half is fused with the amorphous axial intercentrum, which is broader than the axial centrum in anterior view (Fig. 4A), resembling the condition of *Coloradisaurus*^21^. All the centra of the eight postaxial cervical vertebrae are low and elongate, with the corresponding neural arches closely fused to them. Ce3 possesses a relatively larger centrum than the axis, with a length/height ratio approximately 3.6. This ratio continues in the following two cervicals, similar to the condition of most non-sauropodan sauropodomorphs, whereas some massospondylids (e.g. *Coloradisaurus*^21^ and *Leyesaurus*^24^) possess relatively longer centra of anterior vertebrae with a length/height ratio of 4–5. A sagittal ventral keel is developed on every centrum (Fig. 4A), and becomes more prominent from anterior to posterior. The epipophyses are present as low ridges that extend along the dorsal surfaces of the postzygapophyses.

The centra of the dorsal vertebrae are amphicoelous and possess markedly constricted ventral surfaces as in most non-sauropodan sauropodomorphs. The ventral keels are developed on the first three dorsals, but fade away posteriorly. The lateral surfaces of the dorsal centra are strongly concave on D3–D6 at the mid-height (Fig. 4B), but these depressions are imperforate and lack well-defined pleurocoels that present in sauropods. The neural spines of the anterior three dorsals possess transversely widened dorsal summits, a feature that is present in other Lufeng specimens, including *Xingxiulong*^6^ and *Yunnanosaurus*^28^. In lateral view, the posterior margins of the neural spines are straight in D1–D3, whereas the more posterior vertebrae have concave posterior margins with projecting pointed posterodorsal corners; in contrast, *Yunnanosaurus*^28^ and *Jingshanosaurus*^26^ possess straight margins of their posterior neural spines. The parapophyses of the first three dorsals are located anterodorsally to the midpoint of the centra, and progressively move upward until they are entirely placed on the neural arch by D6. The hyposphenes are well-developed in the dorsal vertebrae, with a height that is subequal to that of their respective neural canals in dorsals from the anterior half of the series (Fig. 4B), which is similiar in sauropods, but contrasting with the those are less than the height of the neural canals of most non-sauropodan sauropodomorphs.

The sacrum is composed of three elements. According to their relative position and the closer fusion of the posterior two centra; the first vertebra is identified as a dorsosacral followed by two primordial sacrals (Fig. 4D). The transverse process of the first sacral is smaller than those of the posterior two, in which the expanded transverse processes partially roof the intercostal spaces. The shape of the iliac articular facets for the first sacral rib is singular, whereas the sacral ribs of the two primordial sacrals are partially broken and repaired artificially.

The five anterior-most caudal vertebrae are preserved in LFGT-ZLJ0033. The anterior three vertebrae are well-preserved, while the centra of the following two are fused via a large extra-osseous growth (Fig. 4C). This pathological swelling appears to be due to ossification of the annulus fibrosus^17^. The centra of the anterior three caudals are dorsoventrally tall and anteroposterior short, with a centrum length/height ratio approximately 0.7, which is a common metric observed in sauropodiforms^2^. The ventral surfaces of the anterior caudals bear ventral keels rather than longitudinal furrows.

#### Pectoral girdle

**Figure 5:**
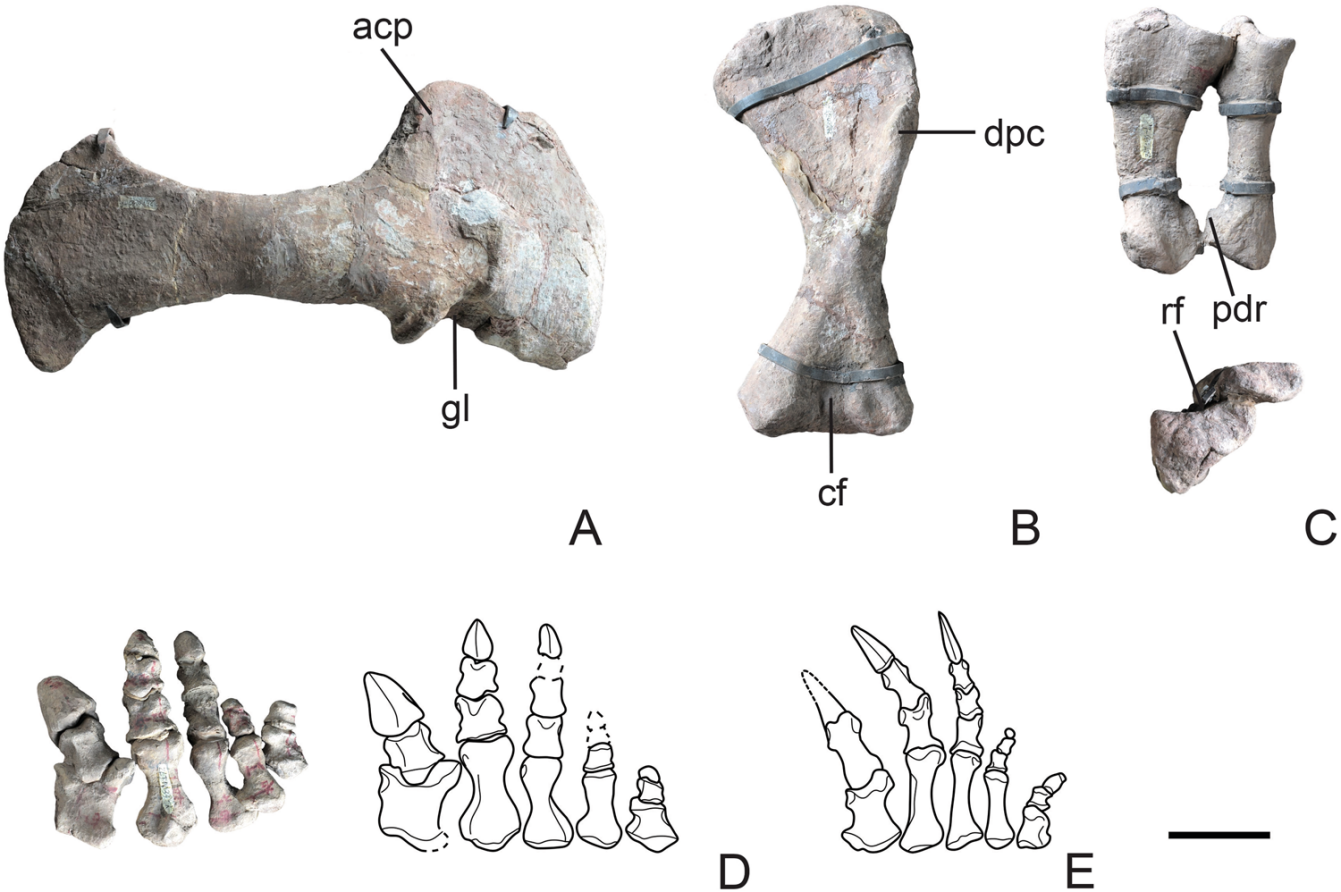
Pectoral girdle and forelimb of Yizhousaurus sunae gen. et sp. nov. (**A**) Right scapulocoracoid in lateral view; (**B**), left humerus in anterior view; (**C**), left ulna and radius in posteromedial and proximal (anterior points up) views; (**D**), photograph and schematic line drawing of right manus in dorsal view; (**E**), schematic line drawing of right manus of *Plateosaurus* (after Huene, 1926a) for comparison. **Abbreviations:** acp, acromion process; cf, cuboid fossa; dpc, deltopectoral crest; gl, scapular glenoid; pdr, posterodistal ridge; rf, radial fossa. Dashed lines represent reconstruction. Scale bar equals 10 cm. (The photographs are taken by Wei Gao, and line drawings are created by Q.-N.Z.)

Both scapulae are complete and fused with their respective coracoids. The midshaft of the scapula is constricted with distinctly expanded dorsal and ventral ends (Fig. 5A). The minimum anteroposterior width of the scapular blade is approximately 18% of the dorsoventral scapular length, which is slightly wider than in many other non-sauropodan sauropodomorphs of which this ratio varies from 15% to 17%^29^; however, the scapular blade is narrower than that of *Jingshanosaurus*^26^ and *Xingxiulong*^6^, in which this ratio is approximately 19~20%. The acromion process is well-developed with a gently curved anterior border, while the dorsal margin abruptly rises from the scapular blade at an angle of approximately 60°. The width of the dorsal expansion is approximately 49% of the total scapular length. The dorsal margin is convex with a well-developed posterodorsal corner. The **coracoid** is a broad, subtriangular plate with its overall morphology resembling that of most non-sauropodan sauropodomorphs. The glenoid facet is ventrally concave and surrounded by a prominent lip (Fig. 5A).

#### Forelimb

The humerus is a stout bone with prominent proximal and distal expansions (Fig. 5B), and is 20% shorter than the scapula. The humeral head is robust and convex proximally. The deltopectoral crest occupies about 45% of the total humeral length, similar to most non-sauropodan sauropodomorphs with this ratio ranging from 45%–55%^30^. The **ulna** is about 60% the length of the humerus, which is similar to most non-sauropodan sauropodomorphs. The proximal end of the ulna is expanded both mediolaterally and anteroposteriorly, resulting in a thick subtriangular profile in dorsal view. A shallow radial fossa, delimited by the blunt anterolateral and anteromedial processes, is present on the proximal end (Fig. 5C). The radius is short and gracile with respect to the ulna, and is about 56% the length of the humerus. Its proximal end is subelliptic and more expanded than the distal end, while the posterior margin of the distal end has a proximodistally orientated ridge that represents the attachment for the radioulnar ligament (Fig. 5C), as also observed in other sauropodiforms (e.g. *Mussaurus*^30^ and *Aardonyx*^31^). The manus of LFGZ-ZLJ0033 is distinctly short, with the total proximodistal length of the first metacarpal approximately 14% of that of the humerus plus radius, a feature that is characteristic of many sauropodiforms (*Jingshanosaurus*^26^, *Yunnanosaurus*^22^, *Mussaurus*^30^, ‘*Melanorosaurus*’^23^ and *Antetonitrus*^1^). Furthermore, the manus is broad and possesses relatively robust digits (Fig. 5D), with the mediolateral width of the non-terminal phalanges almost equal to their proximodistal lengths, similar to the condition of the sauropodiform taxa mentioned above, as well as in *Riojasaurus*^32^, *Lufengosaurus*^16^ and *Aardonyx*^31^, but unlike the condition in most other non-sauropod sauropodomorphs where the phalanges are longer than broad (e.g., *Plateosaurus*, Fig. 5E, after Huene, 1926a).

#### Pelvic girdle

**Figure 6:**
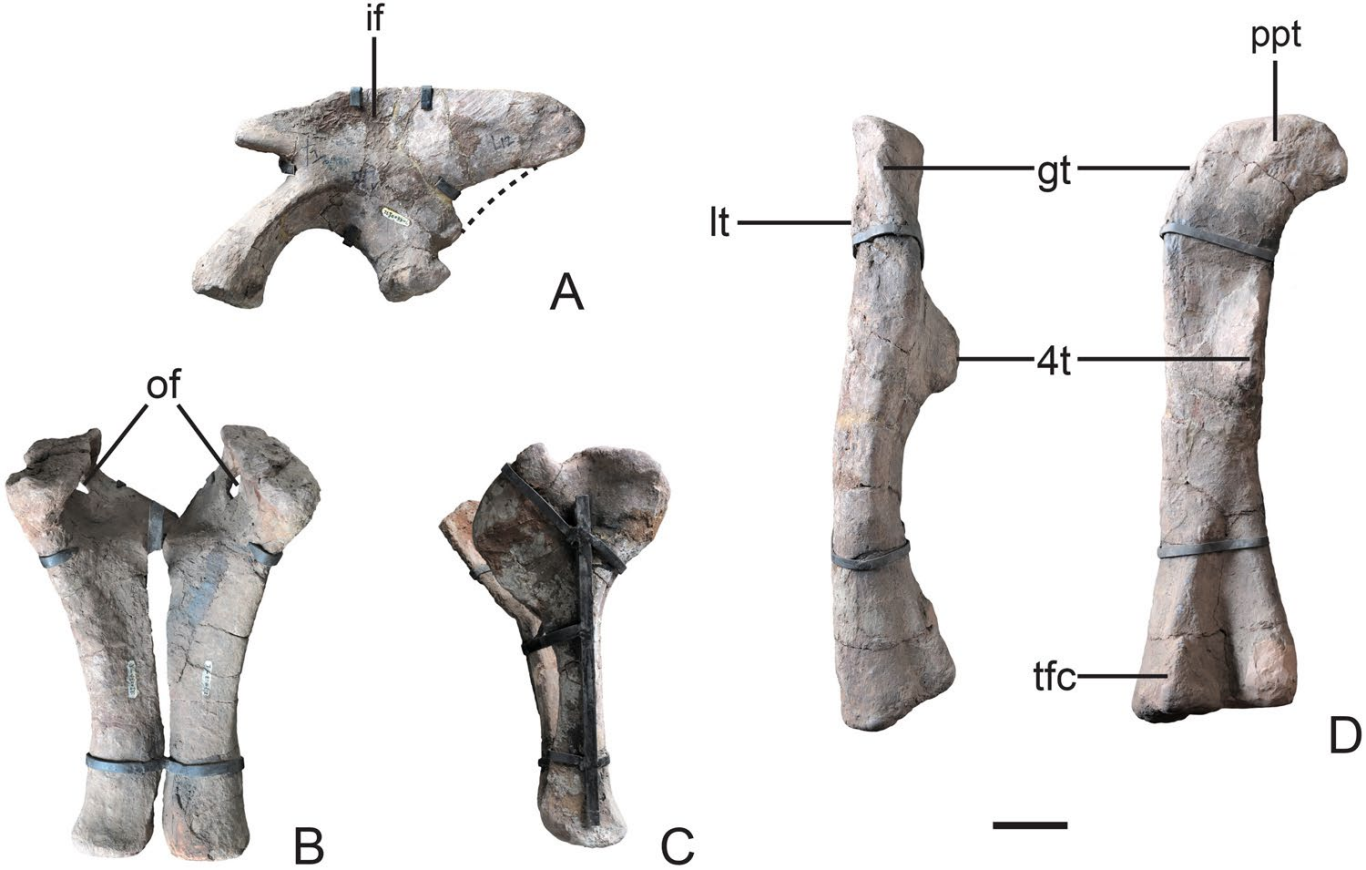
Pelvic girdle and femur of Yizhousaurus sunae gen. et sp. nov. (**A**) Left ilium in left lateral view; (**B**) pubes in anterodorsal view; (**C**) ischia in left lateral view; (**D**) left femur in lateral and posterior views. **Abbreviations:** if, insertion for iliofemoralis muscle; gt, greater trochanter; lt, lesser trochanter; of, obturator foramen; ppt, posteroproximal tubercle; tfc, tibiofibular crest; 4t, fourth trochanter. Dashed lines represent reconstruction. Scale bar equals 10 cm. (The photographs are taken by Wei Gao.)

The pelvic girdle is well-preserved as three separate elements. The **ilium** is low and long, and the preacetabular process is subtriangular, while the ventral margin of the postacetabular process is partially broken (Fig. 6A). The concave area for the iliofemoralis muscle is restricted to the dorsal half of the lateral surface of the iliac plate. The ischial peduncle is slightly shorter than the pubic one, and has a projected ‘heel’ at its posteroventral corner, which is commonly seen in non-sauropodiform sauropodomorphs. The pubic plate is broad and occupies approximately 27% of the total pubic length, with the small obturator foramen visible in anterior view (Fig. 6B). The lateral margin of pubic apron has a concave profile in anterior view, and the distal end of the pubis is distinctly expanded dorsoventrally, with its depth 16% of the proximodistal pubic length. The ischium is slender and shorter than the pubis, with a ‘Y’-shaped profile in lateral view (Fig. 6C). The ischial obturator plate is expanded and occupies the proximal one-third of the ischium. The ischial shaft is rod-like and subtriangular in cross section. Its distal end is moderately expanded with the depth approximately 1.7 times its mediolateral width.

#### Hind limb

The femur is long and straight in both anterior and posterior views, whereas it is weakly sigmoidal in medial and lateral views (Fig. 6D). The proximal end has a well-developed femoral head, with a weakly developed tubercle present on the posterior surface of the proximal end. The greater trochanter is developed as a sinuous ridge in lateral view and situated at the level of the femoral head. The lesser trochanter is present as a straight ridge located on the anterior surface of the femoral shaft, proximodistally extending below the femoral head. The fourth trochanter is well-developed as an elongated crest positioned on the posteromedial margin of the proximal-half of the femur, and is asymmetric in medial view. The cross section of the femoral shaft is subelliptical with the long axis orientated mediolaterally. It is less compressed anteroposteriorly than that of sauropods, but different from the subcircular cross section in most non-sauropodiform sauropodomorphs. The distal condyles are almost equal in size with rounded margins, and the fibular condyle is posteriorly delimited by a tibiofibular crest.

## Discussion

**Figure 7:**
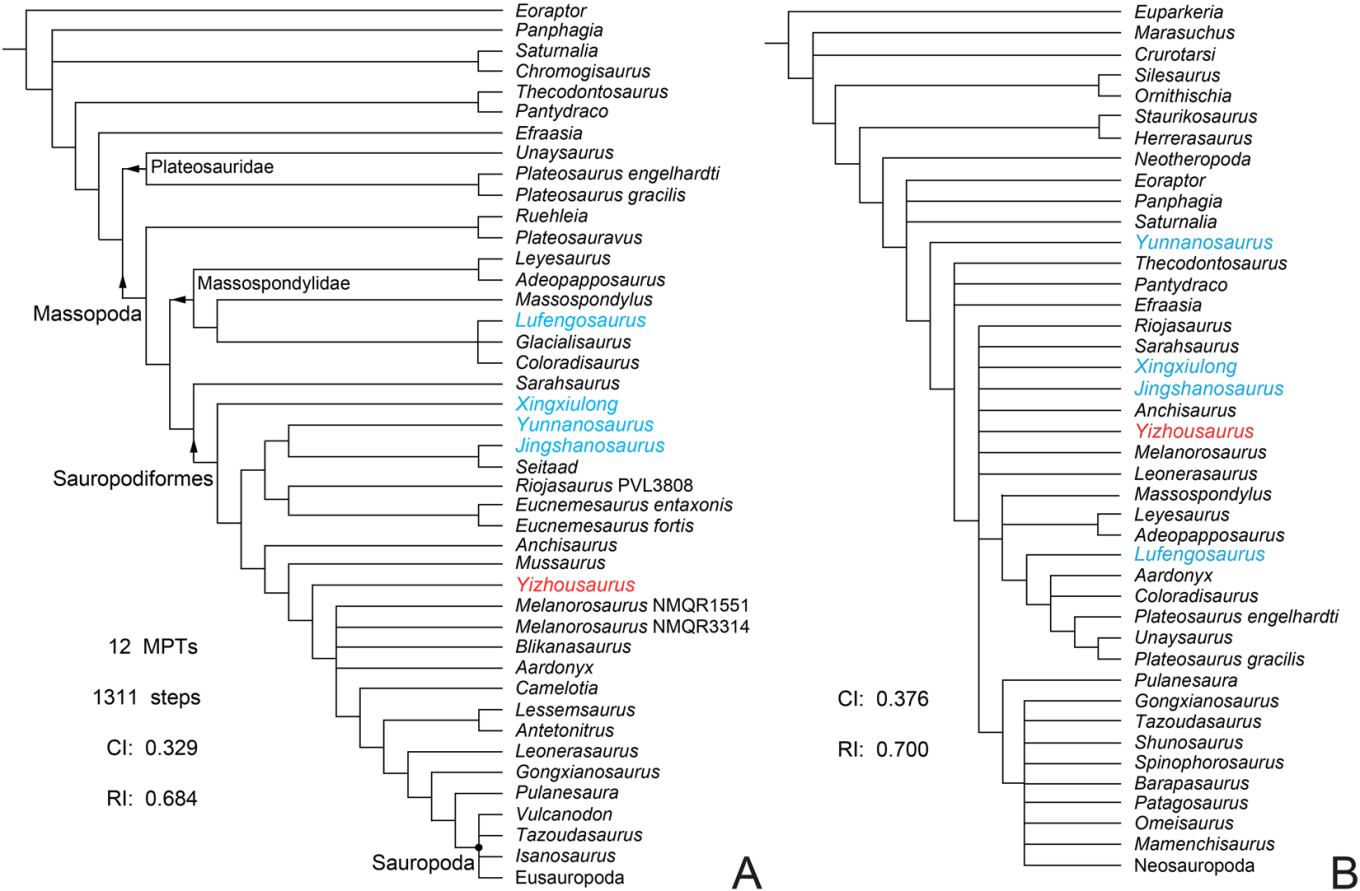
Phylogenetic analyses depicting the position of Yizhousaurus sunae gen. et sp. nov. (**A**) The abbreviated strict consensus tree within Sauropodomorpha, dark dot represents node-based definition, and arrows represent stem-based definitions; (**B**) the strict consensus tree only based on cranial data. *Yizhousaurus* is colored in red, and other materials from Lufeng are colored in blue. **Abbreviations:** CI, consistency index; MPTs, most parsimonious trees; RI, retention index.

In order to investigate the phylogenetic affinity of *Yizhousaurus sunae*, a cladistic analysis is conducted based on the data matrix of McPhee & Choiniere^5^. The definitions of all the clades mentioned in this study follow Wang *et al*.^6^. The strict consensus tree obtained from our phylogenetic analysis is relatively well-resolved (Fig. 7A), with *Yizhousaurus* recovered as a sauropodiform close to *Anchisaurus* and *Mussaurus*. The node [*Yizhousaurus*+] is supported by four postcranial synapomorphies: the ventrolateral twisting of the transverse axis of the distal end of the first phalanx of manual digit one relative to its proximal end is less than 60 degrees (238.1); the non-terminal phalanges of manual digits two and three is as long as wide (244.1); the cross-section of the mid-shaft of the femur is elliptical with the long axis orientated mediolaterally (283.1); the position of proximal tip of the lesser trochanter is distal to the femoral head (288.1).

It is obvious that large parts of sauropodomorph phylogeny have been reconstructed in the absence of well-preserved cranial material, an issue that is especially true for non-sauropodan sauropodiforms. Based on the strict consensus tree, ‘*Melanorosaurus*’ (NMQR 3314) is the only sauropodiform that preserves relatively good cranial material between *Yizhousaurus* and *Shunosaurus* (the latter being a eusauropod). It possesses a typical plateosaurid-like skull in general appearance^23^, while occupying a slightly closer position than *Yizhousaurus* to the root of Sauropoda in the phylogenetic tree. Conversely, the cranium of *Yizhousaurus* displays some ‘sauropodan’ cranial features, (e.g., the reduction of the antorbital fenestra and external mandibular fenestra, and the presence of lateral plates at least on upper jaws), this raises an interesting question as to how to explain the unique characters of *Yizhousaurus*: are these similarities genuine apomorphies, or the result homoplastic convergence? However, the current phylogenetic results might be biased by postcranial information due to the paucity of cranial materials. In order to explore the cranial evolution of sauropodiforms and test the robustness of the total data analysis, herein we performed an alternative cladistic analysis based on a dataset that is pruned of both postcranial characters as well as taxa without any cranial elements. The strict consensus tree of the cranial only dataset recovers a topology with large polytomies, and the affinities of most non-sauropodan sauropodiforms including *Yizhousaurus* and ‘*Melanorosaurus*’ are still unsolved (Fig. 7B). But it is worth noting that *Yunnanosaurus* is recovered outside Sauropodiformes and even Massopoda, and *Aardonyx* falls within a clade containing both plateosaurids and massospondylids. This result suggests an unappreciated amount of cranial homoplasy in non-sauropodan sauropodiforms, providing some interpretative context for the distinctive skull of *Yizhousaurus*. Moreover, the CI and RI values have been calculated for independent cranial and postcranial character partitions (the strict consensus of the postcranial only dataset see Supplementary Information), the results reveal slightly lower inversion of cranial characters. But the cranial homoplasy reflected in individuals may differ from overall taxa, such as those ‘sauropodan’-like cranial characters in *Yizhousaurus* are absent in other closely related groups, which suggests the mosaic evolution in individual characters through sauropodiformes is more interesting and nuanced than generally considered. Nonetheless, it is obvious that more well-preserved cranial material, along with associated post-crania, is required to better understand the series of character transformations that led to the distinctive sauropod skull.

Our phylogenetic analysis also indicates that *Yizhousaurus* is the taxon closest to the base of Sauropoda among all currently known non-sauropodan sauropodomorphs from the Lufeng Formation, and therefore it expands the taxonomic diversity and morphological disparity of Lufeng sauropodomorphs. Besides *Yizhousaurus*, the only other sauropodomorph reported from the Lufeng Formation in the Chuanjie Basin is *Xixiposaurus*^33^. However, we were unable to examine the material of this taxon first-hand, and the current published description, especially regarding the postcranial elements, is too brief for us to reliably code. Nevertheless, it is clear that *Yizhousaurus* can be distinguished from *Xixiposaurus* based on the following differences: *Xixiposaurus* has a much more gracile and smaller overall skeletal architecture than *Yizhousaurus*; the antorbital fenestra and the external mandibular fenastra are larger in *Xixiposaurus* than those of *Yizhousaurus*; the lacrimal of *Xixiposaurus* is anterodorsally inclined, whereas that of *Yizhousaurus* is vertical; the centrum of the fourth cervical is the longest in *Xixiposaurus*, whereas that of Ce7 is the longest in *Yizhousaurus*; and the ‘V’-shaped notch in the posterior edge of the femoral fourth trochanter of *Xixiposaurus* is absent in *Yizhousaurus*.

Four currently uncontroversial non-sauropodan sauropodomorphs from the Lufeng Basin (*Lufengosaurus*, *Yunnanosaurus*, *Jingshanosaurus* and *Xingxiulong*) are included in our cladistics analysis. *Lufengosaurus* and *Yunnanosaurus* were originally described by C. C. Young^28,34^ in 1940s, and their cranial anatomy has been reexamined with amended diagnoses by Barrett *et al*.^16,22^. *Xingxiulong*^6^ was reported with four sacral vertebrae. Hence, it is easy to distinguish *Yizhousaurus* from the above three genera according to their autapomorphies, respectively. However, *Jingshanosaurus* was erected based on a complete skeleton, and was described in Chinese^26^ before the specimen was fully prepared. Morphological features proposed as the diagnostic characters of *Jingshanosaurus* are mostly indistinct, with the majority present in a wide variety of non-sauropodan sauropodomorphs. In fact, many cranial character codings of *Jingshanosaurus* are incorrect due to the restoration on its skull roof. Nevertheless, *Yizhousaurus* can be obviously distinguished from *Jingshanosaurus* based on the following features: the lateral plates are absent in *Jingshanosaurus*; the antorbital fenestra of *Jingshanosaurus* is subtriangular and much larger than that of *Yizhousaurus*; the posterior margin of middle dorsal neural spines of *Jingshanosaurus* is straight in lateral view, whereas that of *Yizhousaurus* is concave; the ischial peduncle of the ilium of *Yizhousaurus* has a projecting ‘heel’ but absent in *Jingshanosaurus*; and the shape of the postacetabular process of the ilium is different in these two taxa.

In addition, another cranium from the Lufeng Formation in the Lufeng Basin was identified as *Jingshanosaurus* cf.^35^, and subsequently was regarded as a new genus *Chuxiongosaurus* by Lü *et al*.^27^. Unfortunately, most of the diagnoses of this taxon are either ambiguous or erroneous, or similar in condition to *Jingshanosurus*, thus its validity and distinctiveness relative to *Jingshanosaurus* requires reevaluation. However, with respect to the differences between *Chuxiongosaurus* and *Yizhousaurus*: the maxillary ascending process of *Yizhousaurus* possesses a pronounced posterodorsal expansion that extends towards the lacrimal rendering the antorbital fenestra pipe-shaped in outline, whereas this expansion is absent in *Chuxiongosaurus* and its antorbital fenestra is subtriangular; the shape of the suborbital region of the jugal in *Yizhousaurus* is an elongate bar, whereas that of *Chuxiongosaurus* is an anteroposteriorly shortened plate; the transverse width of the postorbital ventral process of *Yizhousaurus* is wider than that of *Chuxiongosaurus* relative to the anteroposterior length; the dorsally elevated anterior tip on the dentary of *Yizhousaurus* is absent in *Chuxiongosaurus*; the right external mandibular fenestrae of *Chuxiongosaurus* are obscured by glue, and the left one is broken, but its border can be recognized (pers. observ.), indicating that it has a slightly larger relative size than the same feature in *Yizhousaurus*.

The key points in the transformation to sauropods are generally considered to be linked to a series of complex processes pertaining to body size change, dietary shifts, and the acquisition of a quadrupedal posture^36–38^. As a medium-sized sauropodiform, *Yizhousaurus* represents some apomorphic features of its cranium related to changes in feeding behavior. Regarding the cranium, the height/length ratio of the skull of *Yizhousaurus* is greater than 0.5 (see Supplementary Information). This is greater than that of other non-sauropodan sauropodomorphs; for example, this proportion is around 0.3~0.4 in plateosaurids^19,39^ and massospondylids^16,20,24,40^. Secondly, many supporting bones of the skull are distinctly thick mediolaterally, such as the robust lacrimal, the ventral process of the postorbital, and the quadrate. Thirdly, the reduction of the antorbital fenestra and external mandibular fenestra are consistent with those of sauropods, especially the obliteration of the external mandible fenestra is observed to result in robuster crania^39^. In addition, the existence of the lateral plates on both premaxilla and maxilla possibly braced the teeth against forces resulting from feeding^39–42^, suggesting the piecemeal acquisition of the sauropod cranial bauplan in the early evolution of non-sauropodan sauropodomorphs.

Although the cranial anatomy of *Yizhousaurus* bears some curious similarities (convergent or otherwise) with sauropod dinosaurs, this is contrasted with a postcranial skeleton that retains a ‘prosauropod’-type morphology. However, sauropods possess an integrated suite of modifications relating to forelimb parasagittalism^5,38^, the forelimb of *Yizhousaurus* remains a stocky antebrachium that probably retained a strong degree of flexion at the elbow. Nonetheless, the relatively robust manual morphology of *Yizhousaurus* is close to that of *Antetonitrus*^1^, with the proximal width of the first metacarpal approximately 90% of its total length, whereas this ratio in most non-sauropodiform sauropodomorphs and early sauropods falls within a range of 0.6–0.8^38^. Moreover, the shape of non-terminal manual phalanges is as long as wide in *Yizhousaurus*. The robust, shortened manus of *Antetonitrus* and other derived non-sauropod sauropodiforms has been interpreted as resulting from use of the hand as an auxiliary support structure during locomotion without compromising its primitive grasping capacities, with the manual morphology of *Yizhousaurus* possibly having evolved along similar lines. Furthermore, the straightening of the femoral shaft and the moderately anteroposterior compression observable in *Yizhousaurus* reflects the accommodation of increasing weight and mediolateral stresses at larger body sizes.

In conclusion, *Yizhousaurus*, as a well-preserved sauropodiform, represents another intriguing addition to the Early Jurassic sauropodomorph record, especially regarding its mosaic features spread across the distinctive cranium and relatively plesiomorphic post-crania. It reveals the possibility that the functional mechanics associated with the sauropod cranium may have been selected for in more than one lineage of sauropodomorphs. However, this interpretation rests on whether some of the more ‘sauropodan’-like features of *Yizhousaurus* are apmorphic with regard to the former, or indicative of an independently branching experiment in sauropodan-style bulk browsing. Whatever its precise affinities, the discovery of *Yizhousaurus* helps to enrich our knowledge of non-sauropodan sauropodomorphs, while also hinting the vast amount that remains to be known with respect to the evolution of near-sauropods.

## Methods and Materials

The material of *Yizhousaurus sunae* (LFGT-ZLJ0033) was discovered and excavated by the crews of the Bureau of Land and Resources of Lufeng County from October 20 through November 23 in 2002 from the uppermost bed of the Zhangjiaao Member (Dark Red Beds) Lufeng Formation near the Duwafang Village (Fig. 1). The skeleton was nearly complete and the fossil site had been demarcated into the Lufeng Dinosaur National Geopark. The bones of *Yizhousaurus* were exposed out of the plaster jackets during preparation in the lab using pin vice, brush and pneumatic airscribe to remove the matrix.

In order to determine the phylogenetic position of *Yizhousaurus* within Sauropodomorpha, we performed the phylogenetic analysis which is comprised of 364 characters and 60 taxa (see Supplementary Information) based on the data matrix by McPhee & Choiniere^5^ and analyzed using TNT (ver. 1.1), applying a heuristic search of 1000 replicates of Wagner trees with random addition sequences followed by TBR branch swapping with 10 trees saved per replication, and resulting in 12 MPTs with a shortest length of 1311 steps (Fig. 7A). Characters were equally weighted. The following 43 multistate characters were coded as ordered: 8, 13, 19, 23, 40, 57, 69, 92, 102, 117, 121, 131, 134, 145, 148, 150, 151, 158, 163, 168, 171, 178, 185, 208, 211, 218, 226, 231, 238, 246, 254, 257, 270, 282, 303, 309, 317, 337, 350, 353, 355, 360, 364.

## Electronic supplementary material

Supplementary Information
