# Supplementary material for: A new sauropodiform dinosaur with a ‘sauropodan’ skull from the Lower Jurassic Lufeng Formation of Yunnan Province, China
Source: Sci Rep. 2018 Sep 7;8:13464. doi: 10.1038/s41598-018-31874-9 (PMC6128897; doi:10.1038/s41598-018-31874-9)
Supplement: Supplementary file 1 — Supplementary Information [file 41598_2018_31874_MOESM1_ESM.pdf]

SUPPLEMENTARY INFORMATION

A new sauropodiform dinosaur with a 'sauropodan' skull from the Lower Jurassic Lufeng Formation of Yunnan Province, China

Qian-Nan Zhang[1, 2, 3*], Hai-Lu You[1, 2, 3*], Tao Wang[4], Sankar Chatterjee[5]

[1] *Key Laboratory of Vertebrate Evolution and Human Origins of Chinese Academy of Sciences, Institute of Vertebrate Paleontology and Paleoanthropology, Chinese Academy of Sciences, 142 Xizhimenwai Street, Beijing, 100044, P. R. China.*

[2] *CAS Center for Excellence in Life and Paleoenvironment, 142 Xizhimenwai Street, Beijing, 100044, P. R. China.*

[3] *University of Chinese Academy of Sciences, 19A Yüquan Road, Beijing, 100049, P. R. China.*

[4] *Bureau of Land and Resources of Lufeng County, Yunnan Province, 651299, P. R. China.*

[5] *Museum of Texas Tech University, Lubbock, TX 79409, U. S. A.*

* Corresponding authors (email: zhangqiannan@ivpp.ac.cn; youhailu@ivpp.ac.cn)

1. **Supplementary figure S1**: Skull of *Yizhousaurus sunae* gen. et sp. nov. Close-up of the posterior tooth row of the left maxilla in medioventral view to show the tooth serrations on mesiodistal sides and the labial dental lateral plate (left), and the skull in right lateral view (right) of *Yizhousaurus sunae* gen. et sp. nov. Scale bar in right figure equals 100 mm. (The photographs are taken by Wei Gao.)

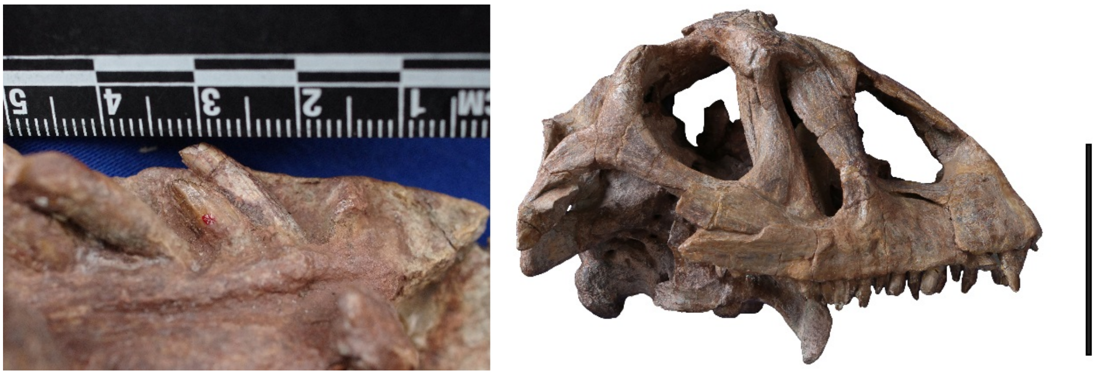

2. **Supplementary figure S2**: Vertebrae of *Yizhousaurus sunae* gen. et sp. nov. A, cervical vertebrae; B, complete dorsal vertebrae; C, anterior caudal vertebrae. Scale bar equals 100 mm. (The photographs are taken by Wei Gao.)

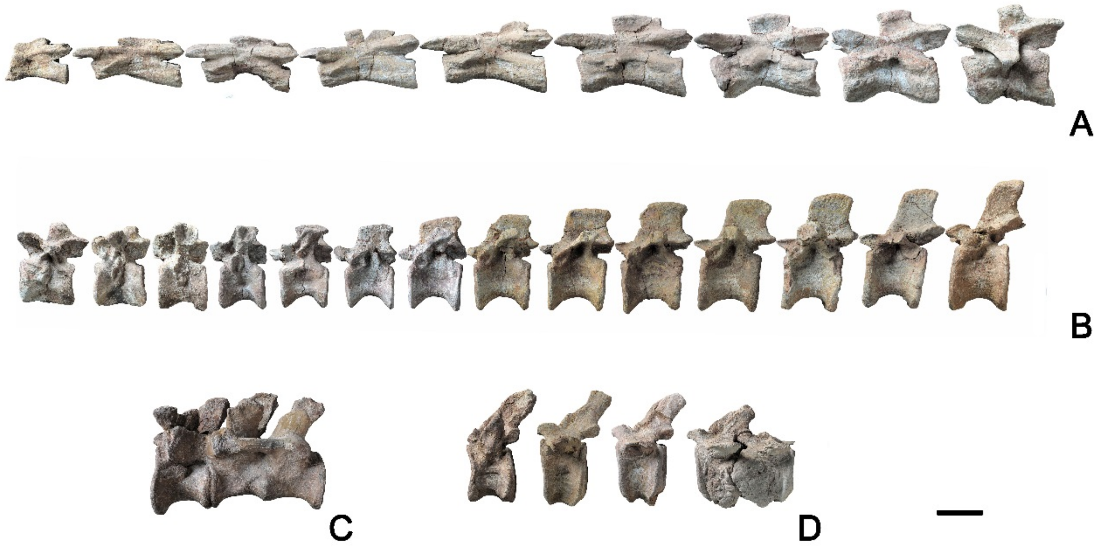

3. **Supplementary figure S3**: Limb bones of *Yizhousaurus sunae* gen. et sp. nov. A. left humerus in anterior, lateral, posterior, medial, proximal and distal views (left to right, respectively); B. left femur in anterior, medial, posterior, lateral, proximal and distal views (left to right, respectively). Scale bar equals 100 mm. (The photographs are taken by Wei Gao.)

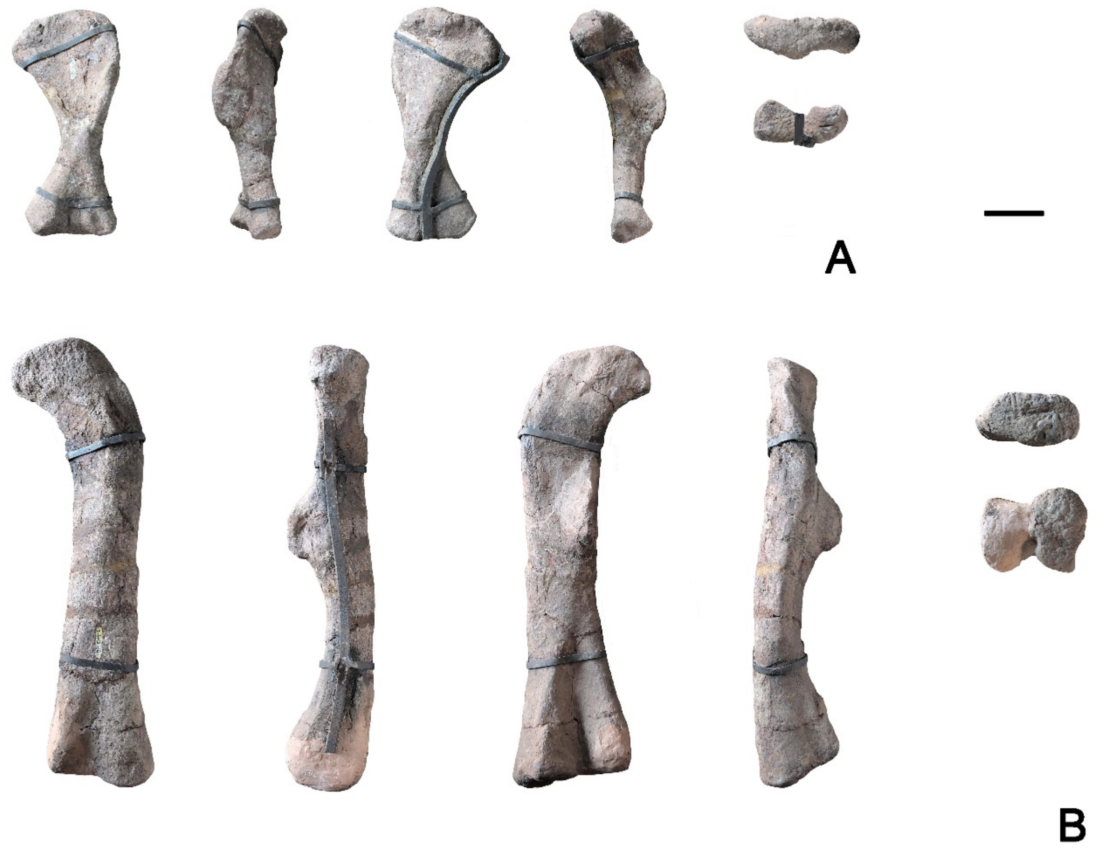

4. **Supplementary figure S4**: The strict consensus trees of the complete dataset (left) and postcranial only dataset (right). Numbers below the nodes represent bootstrap frequencies higher than 50% and Bremer support values higher than 1.

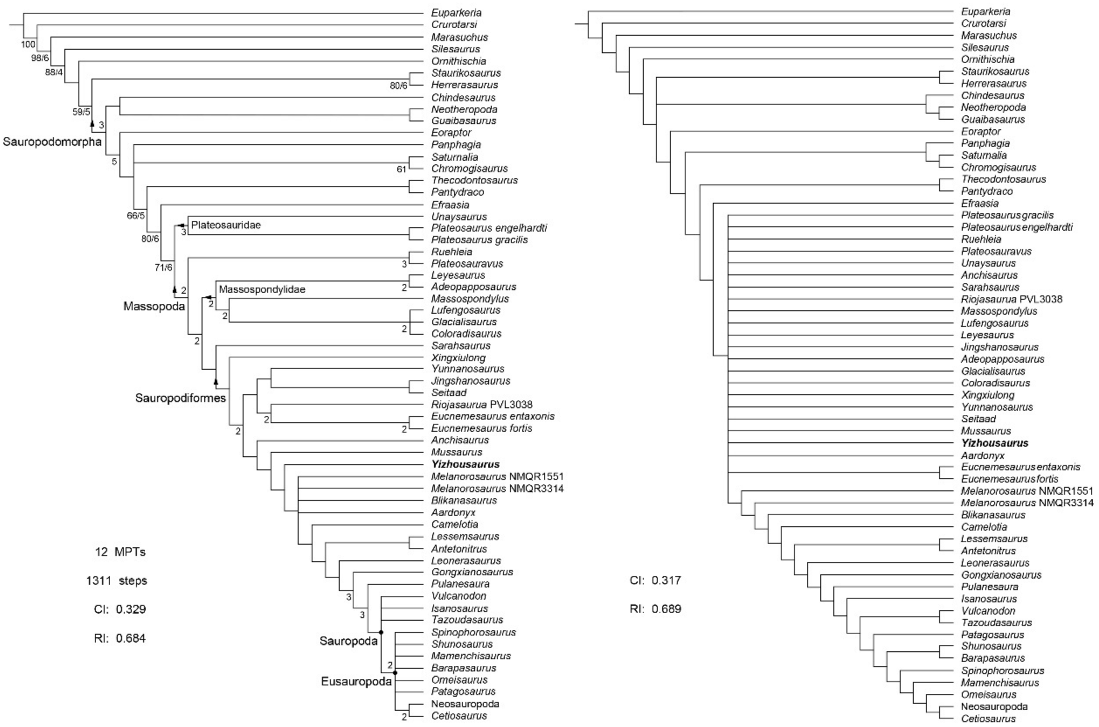

5. Character states of *Yizhousaurus sunae* gen. et sp. nov. used in the phylogenetic analysis in this study.

1101100210	1021111110	1111101111	0110000111	0010001100
1111111100	??00???10?	100?00101?	20?10?0000	0101011001
0??0000110	[0 1]101011001	1?10111100	1000110101	?011010[0 1]00
0011100[1 2]10	0000000010	00010?0100	000110000?	01????????
0010011121	100011110?	?????0010?	2101101101	0001020000
1003110000	0011011002	0110010110	?111011110	0001011110
11????????	??????????	??????????	??????????	??????????
??????????	???[3 4]			

6. **Supplementary Table S1**: Measurements (unit: cm) of the cranium of *Yizhousaurus sunae* gen. et sp. nov. * represents estimated value.

Skull length	26.7*
(the anterior tip of the premaxilla to the posterior margin of the squamosal)	
Skull height	14.9*
(the dorsal margin of the parietal to the ventral margin to the jugal, as both quadrates are disarticulated)	
Skull width (the transverse width across the lateral margins of both postorbitals)	18.6
Maximum diameter of the exernal naris	7.1*
Length of the antorbital fenestra	3.1
Length of the orbit	5.2*
Length of the supratemporal fenestra	3.5
Length of the lower jaw	29.8
Height of the lower jaw	3.9
Maximum diameter of the external mandible fenestra	1.5

7. **Supplementary Table S2**: Measurements (unit: cm) of the postcranial skeleton of *Yizhousaurus sunae* gen. et sp. nov. Abbreviations: AnF, anterior face of the centrum; C, centrum; Ca, caudal vertebrae; Ce, cervical vertebrae; D, dorsal vertebrae; Di, diapophysis (mediolateral length); Hy, hyposphene; Mc, metacarpals; NA, neural arch; NS, neural spine (height: measured from the dorsal margin of the postzygapophysis to the top of the neural spine; length: measured at midspine height anteroposteriorly); Pl, phalanges; PoF, posterior face of the centrum; PoZ, postzygapophyses; PrZ, prezygapophyses; S, sacral vertebrae; TP, transverse processes (mediolateral length). * represents estimated value.

Cervical Vertebrae

Element	Height	C length	C Height	AnF Height	AnF Width	PoF Height	PoF Width	NS Height	NS Length	NS width (top)	PrZ Length	PrZ Width	PoZ Length	PoZ Width
Axis	7.5	12.9	3.8	4.0	4.9	4.3	4.1	2.0	8.2	0.5			4.9	2.9
Ce3	8.8	18.5	5.2	4.2	5.1	5.0	5.2	2.3	9.6	0.7	10.0	2.4	8.8	3.6
Ce4	8.8*	18.5	5.3*	4.8	5.5	5.2	7.1	3.0	10.6	1.3	10.9	3.3	9.9	3.9
Ce5	11.6	20.6	5.7	5.1	6.2	6.5	7.8	3.6	10.9	1.5	11.7	3.7	11.0	4.6
Ce6	12.2*	21.5	6.5	6.2	7.2	7.8	8.8		11.1*	1.7*	12.4	3.8	11.5	4.8
Ce7	15.0	22.1	8.6	7.8	8.0	8.5	9.7	4.1	11.2	1.8	12.9	4.5	12.5	5.2
Ce8	16.2	21.5	8.8	9.4	9.8	8.4	10.8	5.1	9.1	2.0	13.1	4.8	13.0	5.3
Ce9	18.6	20.7	10.0	10.0	9.0	10.7	11.0	5.4	7.8	2.7	11.9	5.2	12.8	5.8
Ce10	20.9	17.2	12.4	10.2	9.6	11.2	11.9	5.7	5.5	3.2	10.8	5.8	10.2	6.0

Dorsal Vertebrae

Element	Height	C length	C Height	AnF Height	AnF Width	PoF Height	PoF Width	NS Height	NS Length	NS Width (top)	Di length	PrZ Length	PrZ Width	PoZ Length	PoZ Width	Hy height
D1	22.9	14.7	11.1	11.5	11.1	11.4	10.8	5.6	5.7	4.6	10.0	8.5	6.2	7.0	3.8	2.6
D2	22.0	14.1	11.9	12.2	9.7	11.3	9.6	5.0	4.5	4.5	9.3	7.8	6.4	4.5	3.2	2.8
D3	20.1	12.5	9.3	11.1	9.5	11.2	9.5	4.9	4.4	4.1	8.1	6.7	6.2	3.8	3.0	2.9
D4	19.4	12.0	7.5	10.6	9.7	10.4	9.6	5.3	5.6	3.6	7.1	5.4	5.5	3.9	3.0	2.7
D5	18.8	12.3	6.9*	10.1	9.2	10.3	9.7	5.5	7.1	2.8	6.3	5.8*	4.3	3.7	3.2	2.3
D6	18.1	12.0	6.7	9.7	9.5	10.1	9.6	5.7	8.3	2.1		4.9	3.7	3.5	2.9	1.9
D7	19.6	12.9	7.6	10.3	9.6	11.3	9.8	7.2	9.6	1.6	6.8	4.1	2.8	3.4	2.6	2.1
D8	20.1	13.0	7.7	11.0	9.6	11.4	10.3	7.5	9.8	1.5	6.7		2.5	4.6	3.0	1.9*
D9	21.2	13.3	8.1	11.3	10.2	12.0	10.8	7.8	9.8	1.6	7.0	4.5	2.5	3.9	2.9	1.5
D10	22.6	13.5	8.3	12.3	10.9	11.8	11.4	8.7	9.8	1.7	7.6	4.2	2.5	4.7	3.1	1.6
D11	23.3	13.5	8.2	12.3	11.4	12.5	12.0	9.0	9.5	1.8	7.6	4.9	2.6	5.5	3.4	
D12	24.2	12.9	8.5	12.3	11.4	12.8	13.7	9.5	9.2	2.0	7.4	5.3	3.0	5.8	3.4	
D13	24.4	12.6	8.8	12.1	13.2	12.7	14.9	10.2	7.8	2.4	6.7*	5.8	3.1		3.8	1.5
D14	26.3	12.1	8.9	13.2	14.8	13.4	14.8	11.1	7.8	3.1	6.6	6.0	3.5	5.7	3.5	

Sacral Vertebrae

Element	Height	C length	AnF Height	AnF Width	PoF Height	PoF Width	Di length	NS Length	NS Height	NS Width (top)	PrZ Width	PrZ Length	PoZ Width	PoZ Length
S1	27.0	12.9	13.0	14.2			6.6*	8.0	11.4	3.2	3.5	6.2		3.4
S2	26.0*	12.8					9.1	9.6	12.5*	2.4		3.8		
S3	25.8	13.9			13.0	12.3	6.8	7.2	11.9*	1.9		3.0*		

Caudal Vertebrae

Element	Height	C length	C Height	AnF Height	AnF Width	PoF Height	PoF Width	TP length	NS Height	NS Length	NS width (top)	PrZ Length	PrZ Width	PoZ Length	PoZ Width
Ca1	27.4	9.8	13.5	13.4	12.5	13.2	12.6	6.0	14.0*	7.9	2.6			4.2	3.3
Ca2	28.7*	9.5	15.0	14.2	13.1	15.5*	12.1	7.8	12.1*	6.0*	2.5	4.9	3.3	4.4	3.3
Ca3	25.9	9.7	11.7	12.8	12.1	14.8*	11.5	7.9	11.7	6.5	1.9	4.7	3.0	3.8	3.1
Ca4				12.8	11.4			6.8	8.8*	6.2*	1.7	4.1	2.9	3.1	2.7
Ca5						12.0*	9.5	6.5				3.9	2.9		

Pectoral Girdles

Element	Total Length	Least width of scapula blade	Length of articular surface with coracoid	Distal height of scapula blade
Left scapula	54.5	9.6	21.9*	26.2
Right scapula	53.3	9.9	22.6*	26.1
Element	Total Length	Total height	Length of articular surface with humerus	Width of articular surface with humerus
Left coracoid	19.0	25.6	11.0	10.1
Right coracoid	17.2	24.7	10.4*	8.8*

Humeri

Element	Total Length	Deltopectoral crest length	Proximal width (mediolateral)	Humeral shaft minimus width	Distal (condyles) width
Left	43.2	19.4*	22.6	8.2	18.3
Right	42.5	19.1*	22.1	7.7	18.1

Ulnas and Radii

Element	Total length	Proximal maximum anteroposterior length	Proximal maximum mediolateral width	Mid-shaft minimum transverse width	Mid-shaft anteroposterior length	Distal (condyles) anteroposterior length	Distal (condyles) maximum transverse width
Left ulna	25.9	8.4	12.7	5.6	3.9	4.2	9.2
Right ulna						3.9*	
Left radius	24.1	4.3	9.2	4.2	3.4	5.0	7.2
Right radius	24.7*	4.3	8.4	4.8	4.0*	5.1	8.1

Metacarpals and Phalanges

Element	Total length (anteroposterior)	Proximal height (dorsoventrally)	Proximal width (mediolateral)	Distal height (dorsoventrally)	Distal width (mediolateral)
Metacarpals					
Left Mc Ⅰ	9.7	7.3	8.6	6.2*	7.2
Left Mc Ⅱ	9.9	6.1	4.2	3.5	4.9
Left Mc Ⅲ	11.3	4.0	5.4	3.0	4.1
Left Mc Ⅳ	9.0	4.5	4.6	3.1	4.1
Left Mc Ⅴ	5.3	4.4	4.5	3.3	4.2
Right Mc Ⅰ	9.3	7.0	7.7*	4.8	8.4

Right Mc Ⅱ	11.1	5.0	4.0	3.1	5.6
Right Mc Ⅲ	10.6	4.3	5.9	2.9	4.2
Right Mc Ⅳ	8.0	4.3	5.0	2.7	3.5
Right Mc Ⅴ	6.3	3.5	3.4	3.3	4.3
Maniphalanges					
Left Pl Ⅰ-1	5.9	5.8	5.8	4.7	5.4
Left Pl Ⅰ-2	14.6*	8.4	5.9	1.5	1.4
Left Pl Ⅱ-1	4.5	4.8	6.5	2.8	4.9
Left Pl Ⅱ-2	3.4	3.7	3.7	2.5	3.5
Left Pl Ⅱ-3	7.3	4.0	3.6	1.5	1.7
Left Pl Ⅲ-1	2.6	2.9	3.2	2.1	2.6
Left Pl Ⅲ-2	2.3	1.7	2.7	2.0	2.4
Left Pl Ⅲ-3	2.0	1.9	2.3	1.5	2.3
Left Pl Ⅲ-4	4.7	2.2	2.4	0.9	1.1
Left Pl Ⅳ-1	2.9	2.8	3.4	2.1	2.8
Left Pl Ⅴ-1	2.0	2.2	2.3	2.1	2.8
Right Pl Ⅰ-1	5.7	4.4	5.7	4.8	5.0

Right Pl Ⅰ-2	14.2	8.5	5.7	1.3	1.4
Right Pl Ⅱ-1	3.8	4.4	5.2	3.3	4.4
Right Pl Ⅱ-2	3.6	3.8	4.3	3.0	3.4
Right Pl Ⅱ-3	7.3	4.1	3.7	1.0	1.3
Right Pl Ⅲ-1	4.3	4.0	3.9	2.9	3.5
Right Pl Ⅲ-2	2.9	3.0	3.9	2.7	3.7
Right Pl Ⅳ-1	2.6	2.8	2.6	2.3	3.0
Right Pl Ⅳ-2	1.9	2.2	2.3	1.9	2.1
Right Pl Ⅴ-1	2.8	2.5	3.4	2.4	2.6

Ilia

Element	Total length (dorsal margin)	Total height	Acetabulum height	Acetabulum length	Height of preacetabular process	Length of preacetabular process	Height of postacetabular process	Length of postacetabular process	Length/width of pubic peduncle	Length/width of ischium peduncle
Left ilium	48.9	29.3	14.5	19.3	8.7*	9.0	11.1	9.8*	11.4	10.3
Right ilium	47.8*	27.6	13.6	21.8	8.6*	10.4	11.9*	9.6*	10.6	10.5*

Pubes

Element	Total length	Length of pubic plate	Width of proximal end (iliac peduncle + acetabulum)	Length of pubic apron	Minimum transverse width of pubic apron	Depth of distal pubic apron expansion	Transvers width of distal pubic apron
Left pubis	58.0	15.6*	29.1	43.9*	9.5	9.1*	12.2
Right pubis	58.8	16.0*	29.3*	44.8	9.9	9.6	11.7

Ischia

Element	Total length	Height of proximal obturator plate	Length of proximal obturator plate	Minimum width of midshaft	Height of distal end
Left ischium	54.7	26.5*	18.7	4.8	11.9
Right ischium	51.7	24.4	15.9	3.8*	10.4

Femurs

Element	Total length	Mediolateral width of proximal end	Maximum proximodistal depth of femoral head	Proximal end to distal margin of 4th trochanter	Proximodistal length of 4th trochanter	Midshaft mediolateral width	Midshaft anteroposterior width	Anteroposterior length of the distal end	Transverse width of the distal end
Left femur	81.5*	18.8	10.9	36.8	15.3	12.8	9.2	16.8	20.4
Right femur	80.6	17.3	9.1	36.7	15.4	11.3	9.9	17.8	19.7
